# Textured-Based Deep Learning in Prostate Cancer Classification with 3T Multiparametric MRI: Comparison with PI-RADS-Based Classification

**DOI:** 10.3390/diagnostics11101785

**Published:** 2021-09-28

**Authors:** Yongkai Liu, Haoxin Zheng, Zhengrong Liang, Qi Miao, Wayne G. Brisbane, Leonard S. Marks, Steven S. Raman, Robert E. Reiter, Guang Yang, Kyunghyun Sung

**Affiliations:** 1 Department of Radiological Sciences, David Geffen School of Medicine, University of California at Los Angeles, Los Angeles, CA 90095, USA; haoxinzheng@ucla.edu (H.Z.); QMiao@mednet.ucla.edu (Q.M.); SRaman@mednet.ucla.edu (S.S.R.); KSung@mednet.ucla.edu (K.S.); 2 Physics and Biology in Medicine IDP, David Geffen School of Medicine, University of California at Los Angeles, Los Angeles, CA 90095, USA; 3 Departments of Radiology and Biomedical Engineering, Stony Brook University, Stony Brook, NY 11794, USA; jerome.liang@stonybrookmedicine.edu; 4 Department of Urology, David Geffen School of Medicine, University of California at Los Angeles, Los Angeles, CA 90095, USA; WBrisbane@mednet.ucla.edu (W.G.B.); lmarks@mednet.ucla.edu (L.S.M.); rreiter@mednet.ucla.edu (R.E.R.); 5 National Heart and Lung Institute, Imperial College London, South Kensington, London SW7 2AZ, UK; g.yang@imperial.ac.uk

**Keywords:** prostate cancer classification, texture analysis, deep learning, convolutional neural network, PI-RADS

## Abstract

The current standardized scheme for interpreting MRI requires a high level of expertise and exhibits a significant degree of inter-reader and intra-reader variability. An automated prostate cancer (PCa) classification can improve the ability of MRI to assess the spectrum of PCa. The purpose of the study was to evaluate the performance of a texture-based deep learning model (Textured-DL) for differentiating between clinically significant PCa (csPCa) and non-csPCa and to compare the Textured-DL with Prostate Imaging Reporting and Data System (PI-RADS)-based classification (PI-RADS-CLA), where a threshold of PI-RADS ≥ 4, representing highly suspicious lesions for csPCa, was applied. The study cohort included 402 patients (60% (*n* = 239) of patients for training, 10% (*n* = 42) for validation, and 30% (*n* = 121) for testing) with 3T multiparametric MRI matched with whole-mount histopathology after radical prostatectomy. For a given suspicious prostate lesion, the volumetric patches of T2-Weighted MRI and apparent diffusion coefficient images were cropped and used as the input to Textured-DL, consisting of a 3D gray-level co-occurrence matrix extractor and a CNN. PI-RADS-CLA by an expert reader served as a baseline to compare classification performance with Textured-DL in differentiating csPCa from non-csPCa. Sensitivity and specificity comparisons were performed using Mcnemar’s test. Bootstrapping with 1000 samples was performed to estimate the 95% confidence interval (CI) for AUC. CIs of sensitivity and specificity were calculated by the Wald method. The Textured-DL model achieved an AUC of 0.85 (CI [0.79, 0.91]), which was significantly higher than the PI-RADS-CLA (AUC of 0.73 (CI [0.65, 0.80]); *p* < 0.05) for PCa classification, and the specificity was significantly different between Textured-DL and PI-RADS-CLA (0.70 (CI [0.59, 0.82]) vs. 0.47 (CI [0.35, 0.59]); *p* < 0.05). In sub-analyses, Textured-DL demonstrated significantly higher specificities in the peripheral zone (PZ) and solitary tumor lesions compared to the PI-RADS-CLA (0.78 (CI [0.66, 0.90]) vs. 0.42 (CI [0.28, 0.57]); 0.75 (CI [0.54, 0.96]) vs. 0.38 [0.14, 0.61]; all *p* values < 0.05). Moreover, Textured-DL demonstrated a high negative predictive value of 92% while maintaining a high positive predictive value of 58% among the lesions with a PI-RADS score of 3. In conclusion, the Textured-DL model was superior to the PI-RADS-CLA in the classification of PCa. In addition, Textured-DL demonstrated superior performance in the specificities for the peripheral zone and solitary tumors compared with PI-RADS-based risk assessment.

## 1. Introduction

Multi-parametric MRI (mpMRI) acquires anatomical and functional information to assess the aggressiveness of prostate cancer (PCa) [1] and 3T mpMRI has been integrated into guidelines for the diagnosis of PCa [2,3]. The current standardized scheme for the interpretation of mpMRI is the Prostate Imaging Reporting and Data System version 2.1 (PI-RADS v2.1) [4]. PI-RADS has been widely adopted, and studies have shown increased diagnostic performance and superior results in the detection of clinically significant PCa (csPCa) than systematic transrectal US-guided biopsies [5,6,7,8]. However, PI-RADS requires a high level of expertise and exhibits a significant degree of inter-reader and intra-reader variability [9], likely reflecting inherent ambiguities in the classification scheme. Moreover, it is potentially inadequate to rely solely on PI-RADS to fully determine the severity of PCa [9,10]. In particular, several studies reported that only 15% to 35% were biopsy positive among the PI-RADS score 3 lesions when identifying csPCa [11,12,13].

Image texture analysis [14,15] provides the spatial arrangement of intensities in the image and can be used to quantitatively describe the tumor heterogeneity, which can be the primary feature of csPCa [16]. An automated classification of PCa using texture analysis [17] may overcome the current challenges associated with PI-RADS but commonly suffers from the laborious handcrafted feature design process to fully capture the underlying image texture. Alternatively, with the development of deep learning in medical imaging [18,19,20], convolutional neural networks (CNNs) with texture analysis [21] may further improve the accuracy of PCa classification without handcrafted feature engineering.

In this study, we designed a texture-based deep learning (Textured-DL) model for automated PCa classification of suspicious prostate lesions on a 3T mpMRI dataset with whole-mount histopathology (WMHP) correlation. After a lesion was detected and contoured as part of the clinical interpretation, the proposed deep learning model was developed to further improve the classification of PCa for any positive MRI findings (PI-RADS score ≥ 3). The model performance was tested by an independent testing set and compared with the conventional deep learning and PI-RADS-based classification (PI-RADS-CLA) [9,10]. We also conducted the sub-analysis of Textured-DL on lesions with different locations and types (solitary and multi-focal) compared with PI-RADS-CLA.

## 2. Materials and Methods

### 2.1. Study Population and MRI Datasets

With approval from the institutional review board (IRB), this retrospective study was carried out in compliance with the United States Health Insurance Portability and Accountability Act (HIPAA) of 1996. A total of 402 patients who later underwent robotic-assisted laparoscopic prostatectomy (RALP) between October 2010 and June 2018 were enrolled in this study. Detailed characteristics of the patients and tumors are shown in Table 1. Pre-operative prostate mpMRI scans were acquired using a standardized protocol based on the recommendation from PI-RADS. Specifically, the MRI protocol included axial T2 weighted image (T2W) turbo spin-echo (TSE) imaging (repetition time (TR) = 3800–5040 ms, echo time (TE) = 101 ms, field of view (FOV) = 20 cm, matrix size = 320 × 310, in-plane resolution = 0.6 mm × 0.6 mm, slice thickness = 3 mm) and echo-planar diffusion-weighted imaging (EP-DWI) (TR = 3300–4800 ms, TE = 60–80 ms, FOV = 26 cm × 21 cm, matrix size = 160 × 94, in-plane resolution = 1.6 mm × 1.6 mm, slice thickness = 3.6 mm). The apparent diffusion coefficient (ADC) maps were calculated by using linear least squares curve fitting of pixels (in log scale) in the four diffusion-weighted images against their corresponding b values (0/100/400/800 s/mm^2^).

As part of the clinical quality control process, supervised by a senior genitourinary (GU) radiologist (S.S.R.) and urologist (R.E.R.), the fellowship-trained GU radiologists (each had interpreted 1000–3000 prostate mpMRI scans with 10+ years of experience) identified suspicious prostate lesions on the mpMRI. Each lesion was contoured with an assigned PI-RADS score by the radiologists. For MRI scans interpreted before the adoption of PI-RADS v2 (2010–2015), the abdominal imaging fellows and the fellowship-trained GU radiologists, supervised by senior GU radiologist (S.S.R.), retrospectively reviewed and assigned a PI-RADS v2 score to each ROI, blinded to the pathological findings and clinical information at the time of the interpretation. Any lesions with PI-RADS score ≥ 3 were reported as positive findings.

Blinded to MRI, two GU pathologists (each had interpreted up to 1000 prostate wholemount histopathologic reports) identified and outlined tumors on WMHP following RALP as part of the standard of care. On each section, individual PCa lesion size, location, and Gleason Score (GS) (primary and secondary Gleason grade) were reported. Next, at a separate monthly meeting, a multidisciplinary research team consisting of GU radiologists, GU pathologists, and urologists (W.G.B. and R.E.R.) reviewed each case to match the pathologically detected lesion with its corresponding lesion on mpMRI through visual co-registration. Each lesion detected by mpMRI was defined as a true-positive if it corresponded to the same quadrant (left, right, anterior, or posterior) and level (base, midgland, or apex) as the lesion from WMHP; otherwise, it was defined as a false positive (FP) if no corresponding lesions existed on WMHP. False negatives were lesions from WMHP that lacked a corresponding lesion on mpMRI. The index tumor was defined as the most extensive tumor area in the surgical specimen, more specifically, the lesion with the highest GS or the largest diameter when multiple lesions had the same GS. csPCa was defined as a lesion with GS ≥ 3 + 4. After the meeting, all csPCa, indolent lesions (GS = 3 + 3), and FPs were identified based on their zonal and sector anatomy and retrospectively contoured on T2W and ADC images using OsiriX (Pixmeo SARL, Bernex, Switzerland). The ADC images were registered to T2W images by using a non-rigid multimodal registration method [22], which was based on a symmetric non-parametric registration framework in which the sum of squared differences (SSD) of the modality-independent neighborhood descriptor of the images served as the similarity metric, with the Gauss–Newton method as the optimizer. Table 1 summarizes the overall patient and lesion characteristics, stratified by GS, PI-RADS, prostate zones, and lesion focality.

### 2.2. Textured-DL Model

**Figure 1:**
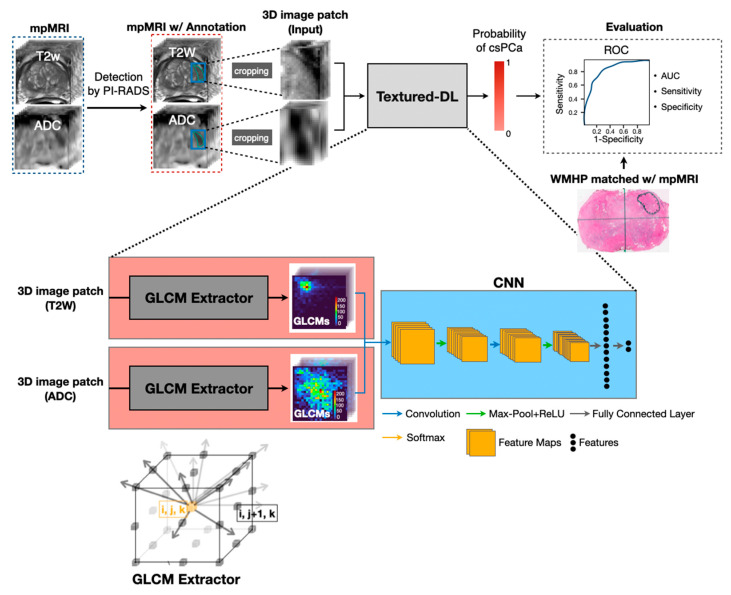
The overall workflow of the proposed Textured-DL model for the PCa classification. Suspicious prostate lesions were first detected and scored by the PI-RADS, followed by contouring. Then, 3D volumetric patches of the prostate lesion were cropped from the T2W and ADC images, and GLCM were extracted from two patches. Next, the two GLCMs were concatenated and fed into CNN to generate the probability of csPCa. ROC curve, AUC, sensitivity, and specificity were adopted to evaluate and compare the performance of PCa classification by the PI-RADS-CLA and Textured-DL, which was confirmed by the histopathological findings. In the GLCM extractor, each cubic box represents a voxel. The distance between adjacent voxels was enlarged to see the directions between voxels clearly. (*i*, *j*, *k*) is a voxel coordinate, and (*i*, *j* + 1, *k*) is an immediate neighboring voxel (INV) coordinate around the voxel.

Figure 1 shows the overall workflow of the proposed Textured-DL model, consisting of a 3D gray-level co-occurrence matrix (GLCM) extractor and a CNN. As part of the clinical MRI interpretation, a suspicious prostate lesion (positive MRI finding (PI-RADS score ≥ 3)) was identified and contoured. Then, the volumetric patches that closely surround the lesion on T2W and ADC were cropped and normalized to 0–255 as the input to Textured-DL. In the Textured-DL, a 3D GLCM extractor was used to extract the 3D GLCM from each volumetric patch (T2W and ADC), and then the 3D GLCMs were concatenated as an input to the CNN. Finally, the Textured-DL outputted the probability that the suspicious prostate lesion was classified as a csPCa.

#### 2.2.1. 3D GLCM Extractor

A prostate lesion volumetric patch was discretized into 64 gray-level bins, yielding a 3D gray-level image with voxel values ranging from 1 to 64. Next, we generated the 3D GLCM by calculating the frequency of voxel pairs with different spatial orientations and specific gray-level values. Unlike the 2D GLCM, which only considers the in-plane pixel adjacency, 3D GLCM also considers the through-plane voxel adjacency. 3D GLCM was calculated as follows:

(1) $$GLCM\left(x,y\right)=\overset{I}{\underset{i}{\sum }}\overset{J}{\underset{j}{\sum }}\overset{K}{\underset{k}{\sum }}\{\begin{array}{c} 1\;\;\;\;\;if\;f\left(i,j,k\right)=x\;and\;f\left(i+di,j+dj,k+dk\right)=y\;\;\;\;\;\;\\ 0\;\;\;\;\;\;\;\;\;\;\;\;\;\;\;\;\;\;\;\;\;\;\;\;\;\;\;\;\;\;\;\;\;\;\;\;\;\;Otherwise\;\;\;\;\;\;\;\;\;\;\;\;\;\;\;\;\;\;\;\;\;\;\;\;\;\;\;\;\;\;\;\;\;\;\;\;\;\;\;\;\;\;\;\;\;\;\;\;\;\;\;\;\;\;\;\;\; \end{array}$$

where *x* and *y* range from 1 to the number of grey levels (*n* = 64), f is the prostate lesion volumetric patch, (i, j,k) is a voxel coordinate, and (i+di,j+dj,k+dk) is an INV coordinate around the voxel. Figure 1 (GLCM Extractor) showed 13 INVs around the (*i*, *j*, *k*) in the 3D space, distributed in 13 directions (one gray arrow represents a direction). One GLCM was produced along each direction. Finally, two sets of GLCMs were obtained on both T2W and ADC.

#### 2.2.2. CNN Network

Two sets of 3D GLCMs for T2W and ADC were concatenated and then fed to the CNN, which consists of two convolutional layers with kernel sizes of 3 × 3 and stride of 1, two pooling layers with a filter size of 2 × 2, and two fully connected layers, to perform the classification of csPCa and non-csPCa. The input and output channel sizes are (26, 32) and (32, 64) for the first and second convolutional layer, respectively. Each convolutional layer was equipped with batch normalization (BatchNorm) and Rectified Linear Unit (ReLU).

### 2.3. Model Development and Comparison

All the deep learning networks were implemented using PyTorch [23]. A 3D GLCM extractor was written using MATLAB. All the training and testing were performed on a desktop computer with a 64-Linux system with Titan Xp GPU with 12 GB GDDR5 RAM. The patient cohort was randomly split into three sub-datasets, including training (*n* = 239; 60%), validation (*n* = 42; 10%), and testing (*n* = 121; 30%) datasets. The model was trained using the training dataset, and hypermeter tuning and best model selection were performed on the validation dataset. Weighted cross-entropy was used as the loss function, which was optimized by the Adam optimizer [24] with the default parameters ($\beta_{1}=0.9$ and $\beta_{2}=0.999$). The learning rate was set to 10^−5^ with a momentum of 0.9. The model was trained for 200 epochs with a batch size of 10.

We used PI-RADS-CLA by the expert reader as a baseline to compare classification performance with Textured-DL. In the PI-RADS-CLA, a PI-RADS score cutoff of 4 was adopted [10,11] to differentiate csPCa from non-csPCa. In assessing the spectrum of PCa found on WMHP, a PI-RADS score of 4 was the most reproducible cutoff for csPCa [10]. Additionally, it has been shown that readers benefit in a statistically significant manner from the deep learning-based method when a threshold of PI-RADS score ≥ 4 is used [25]. In addition, we took the CNN structure from the Textured-DL as another baseline method (Imaged-DL). Moreover, we compared the Textured-DL with a texture feature-based random forest (RF) classification model (Textured-RF) [26]. Within each prostate lesion volumetric patch, the correlation, contrast, homogeneity, and energy on each 3D GLCM for each direction (13 directions in total for each 3D GLCMs) were calculated using scikit-image [27], yielding 104 GLCM-based texture features in total, which were fed to an ensemble learning method, RF [28], for the PCa classification. The RF model was implemented using scikit-learn [29], where the number of the trees was 50, the quality of a split was measured by “gini”, nodes were expanded until all leaves were pure, and bootstrap samples were adopted when building trees. Furthermore, we compared the Textured-DL with two deep CNNs that were previously used for PCa classification. The first one [30] was built on a deep learning model with transfer learning (DTL) [31], and the second one was a relatively deep CNN (DCNN) [32] inspired by VGG-Net [33].

Since lesions with a PI-RADS score of 3 are variable in the diagnosis of PCa, we conducted the sub-analysis of the lesions with a PI-RADS score of 3 compared to lesions with a PI-RADS score of 4–5 for the performance of Textured-DL to diagnose the PCa. In addition, we performed the sub-analyses on the classification of PCa lesions on different prostate zones, such as the peripheral zone (PZ) and transition zone (TZ), and on the index types (solidary and multi-focal lesions) between Textured-DL and PI-RADS-CLA. There exist significant differences in morphological appearance and cancer prevalence between tumors in PZ and TZ, and the assignment of the PI-RADS score for each lesion utilizes different imaging sequences according to zonal anatomy [34]. The aggressiveness of the index tumor is clinically important for treatment decisions, pre-biopsy planning, and pre-surgical planning.

### 2.4. Statistical Analysis

All models (PI-RADS-CLA, Imaged-DL, Textured-RF, DCNN, and Textured-DL) were evaluated on the testing dataset using the area under the ROC (AUC) curve, sensitivity, and specificity. The 95% confidence interval (CI) of the AUC was computed by bootstrapping with 1000 samples. The Wald method [35] was used to calculate the CI of the sensitivity and specificity. The model sensitivity and specificity were selected by the Youden index [36]. Statistical significance was defined as a *p*-value < 0.05. DeLong test [37] was used to perform the AUC comparisons between the baseline methods and the proposed Textured-DL. *p*-values for statistical comparisons of sensitivity and specificity were provided by Mcnemar’s test [38].

## 3. Results

### 3.1. Model Performance in Comparison with PI-RADS-CLA for All Tumors

**Figure 2:**
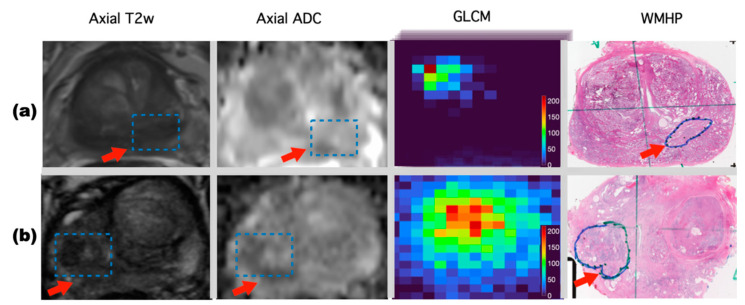
Two examples of prostate lesion classifications are shown in rows (**a**,**b**), respectively. From left to right, axial T2W, axial ADC, GLCMs derived from the prostate lesion volumetric patch and matched WMHP are shown in each row. (**a**) Imaging for a 56-year-old man with a PSA of 12.2 ng/mL. A lesion (blue rectangular box pointed by a red arrow) with a PI-RADS score of 4 and GS 3 + 3 was shown on both the axial T2W and ADC images. The proposed Textured-DL predicted this lesion as a non-csPCa. (**b**) Imaging for a 72-year-old man with a PSA of 8.8 ng/mL. A lesion (blue rectangular box pointed by a red arrow) with a PI-RADS score of 3 and GS 4 + 3 was shown on both the axial T2W and ADC images. The Textured-DL predicted this lesion as a csPCa.

Figure 2 displays two representative examples of mpMRI findings matched with WMHP and predictions of the PCa classification using Textured-DL. Figure 2a represents the imaging for a 56-year-old man with a serum prostate-specific antigen (PSA) of 12.2 ng/mL. A lesion with a PI-RADS score of 4 and GS 3 + 3 was shown on both MRI and WMHP images. The Textured-DL predicted the lesion as a non-csPCa, while this would have been considered as csPCa with PI-RADS-CLA. Figure 2b represents the imaging for a 72-year-old man with a PSA of 8.8 ng/mL. A lesion with a PI-RADS score of 3 and GS 4 + 3 was shown on both MRI and WMHP images. Similarly, Textured-DL predicted correctly, which would have been missed with PI-RADS-CLA.

**Figure 3:**
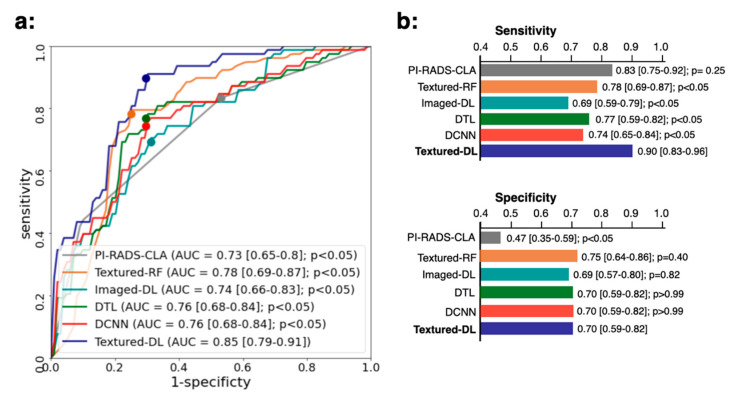
Comparisons of ROC (**a**), sensitivity (**b**), and specificity (**b**) between PI-RADS-CLA, Textured-RF, Imaged-RF, DTL, DCNN, and Textured-DL on the classification of PCa in the overall tumor lesions. CIs are provided in the square brackets. *p* values for the comparison between the baseline methods and the Textured-DL are also shown.

Figure 3 shows the overall classification performance comparison between PI-RADS-CLA, Textured-RF, Imaged-DL, DTL, DCNN, and Textured-DL. For all lesions, Textured-DL demonstrated an AUC of 0.85 (CI [0.79, 0.91]), significantly higher than the PI-RADS-CLA (AUC of 0.73 (CI [0.65, 0.80]); *p* < 0.05), Textured-RF (AUC of 0.78 (CI [0.69, 0.87]); *p* < 0.05), Imaged-DL (AUC of 0.74 (CI [0.66, 0.83]); *p* < 0.05), DTL (AUC of 0.76 (CI [0.68, 0.84]); *p* < 0.05), and DCNN (AUC of 0.76 (CI [0.68, 0.84]); *p* < 0.05). The Textured-DL also demonstrated a significantly higher sensitivity than Textured-RF, Imaged-DL, DTL, and DCNN (all *p* values < 0.05), with a comparable specificity to Textured-RF, Imaged-DL, DTL, and DCNN (*p* values were 0.40, 0.82, >0.99, and >0.99) and a significantly higher specificity than PI-RADS-CLA (*p* value < 0.05).

### 3.2. Classification Performance for Tumors on Different Prostate Zone

**Figure 4:**
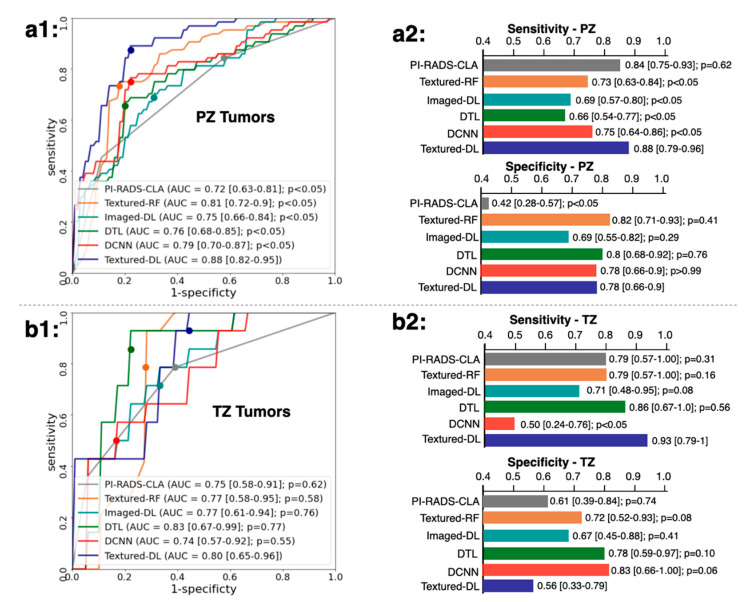
Comparisons of ROC, AUC, sensitivity, and specificity between the Textured-DL and baseline methods in the tumor lesions on the PZ (**a1**,**a2**) and TZ (**b1**,**b2**). CIs are provided in the square brackets. *p* values for the comparison between the baseline methods and the Textured-DL are also shown.

We further conducted the secondary analysis on the different lesion locations, such as PZ and TZ, for the classification performance (Figure 4). In PZ, we found that our Textured-DL achieved an AUC of 0.88 (CI [0.82, 0.95]), significantly higher than PI-RADS-CLA (AUC of 0.72 (CI [0.63, 0.81]); *p* < 0.05), Textured-RF (AUC of 0.81 (CI [0.72, 0.90]); *p* < 0.05), Imaged-DL (AUC of 0.75 (CI [0.66, 0.84]); *p* < 0.05), DTL (AUC of 0.76 (CI [0.68, 0.85]); *p* < 0.05), and DCNN (AUC of 0.79 (CI [0.70, 0.87]); *p* < 0.05). The specificity of Textured-DL (0.78 (CI [0.66, 0.90])) was significantly higher than PI-RADS-CLA (specificity of 0.42 (CI [0.28, 0.57]); *p* < 0.05). Besides, Textured-DL demonstrated a sensitivity of 0.88 (CI [0.79, 0.96]), which was significantly higher than Textured-RF (sensitivity of 0.88 (CI [0.79, 0.96])), CNN (sensitivity of 0.69 (CI [0.57, 0.80])), DTL (sensitivity of 0.66 (CI [0.54, 0.77])), and DCNN (sensitivity of 0.75 (CI [0.64, 0.86])) (all *p* values < 0.05). In TZ, Textured-DL achieved a similar AUC and specificity compared to other baseline methods (PI-RADS-CLA, Textured-RF, Imaged-DL, DTL, and DCNN). The sensitivity of Textured-DL, however, was similar to all other baseline methods except DCNN.

### 3.3. Classification Performance for Solidary and Multi-Focal Tumors

**Figure 5:**
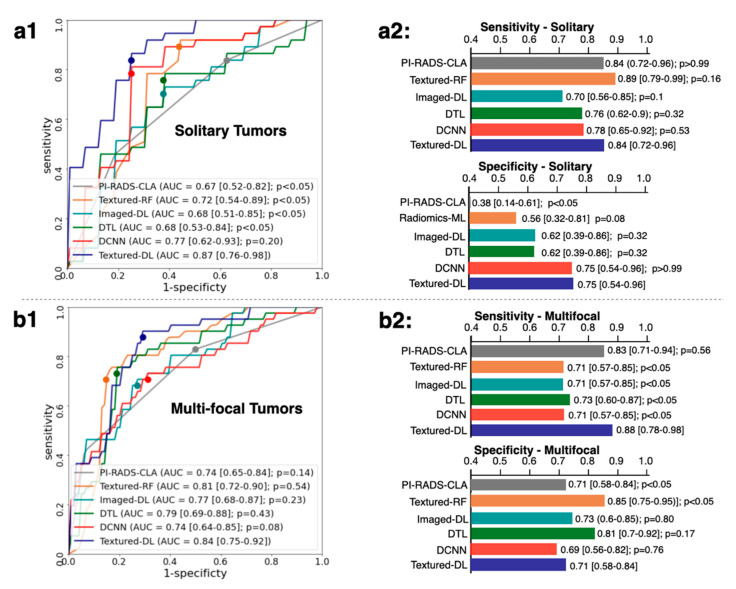
Comparisons of ROC, AUC, sensitivity, and specificity between the Textured-DL and baseline methods in the solitary (**a1**,**a2**) and multi-focal tumors (**b1**,**b2**). CIs were provided in the square brackets. *p* values for the comparison between the baseline methods and the Textured-DL were also provided.

Figure 5 shows another secondary analysis with different tumor types, solitary, and multi-focal tumors. In solitary tumors, Textured-DL demonstrated a significantly higher AUC than all the baseline methods except the DCNN. For the sensitivity and specificity, Textured-DL exhibited a similar result to all other baseline methods except the PI-RADS-CLA in specificity. In multi-focal tumors, Textured-DL achieved a similar AUC to all other baseline methods. The specificity of Textured-DL was significantly higher than PI-RADS-CLA, significantly lower than Textured-RF, and similar with all other baselines. Textured-DL demonstrated a sensitivity similar to that of the other baseline methods.

### 3.4. Classification Performance for Tumors of Different PI-RADS Scores

We also performed the sub-analysis on the classification performance of Textured-DL in the lesions with PI-RADS scores of 3, 4, and 5 (Table 2). We found that the proposed Textured-DL achieved consistent classification performance in AUC, sensitivities, and specificities across different PI-RADS-categorized lesions. AUC and sensitivity for lesions with PI-RADS scores of 4 or 5 were slightly higher than those with a PI-RADS score of 3.

### 3.5. Classification Performance for Index Tumors

We further carried out the sub-analysis with the index tumor lesions only (Table 3). The index tumors were divided into three groups according to the PSA values (PSA < 4, 4 ≤ PSA < 10, and 10 ≤ PSA). For the group of index tumors with PSA < 10 (i.e., low-to-average risk group), Textured-DL achieved a higher sensitivity in detecting csPCa than PI-RADS-CLA, while Textured-DL achieved a better specificity for the group of index tumors with PSA ≥ 4 (i.e., average-to-high risk groups).

## 4. Discussion

A novel Textured-DL method for the PCa classification was proposed by combining CNN with texture analysis [21]. Compared with conventional image texture analysis and deep learning, Textured-DL utilized the spatial arrangement in the MRI signal intensities, which can be used to describe the tumor heterogeneity. Textured-DL has the potential to achieve a better classification performance than conventional CNN and PI-RADS-CLA.

The training and testing for the model were based on the patient cohort who underwent 3T mpMRI prior to radical proctectomy. Although the testing dataset contained similarly distributed lesions (78 csPCa vs. 64 non-csPCa), the results may not be directly translatable for the biopsy planning patient cohort, including biopsy naïve and prior negative biopsy patients, due to lower rates of csPCa. However, our findings in the PI-RADS-CLA were consistent with those of the previous multi-center, multi-reader study [39], and the proposed model consistently achieved higher sensitivities and specificities than the PI-RADS-CLA. We believe that the proposed model can be adopted as an additional means to reduce the overdiagnosis of csPCa in conjunction with radiologists. Future studies, including the biopsy planning cohort for model testing, will further solidify our findings.

The clinical significance of lesions with a PI-RADS score of 3 is considered to be equivocal. The range of positive biopsy rates in lesions with a PI-RADS score of 3 is between 15% and 35% [12,13]. Our method achieved an AUC of 0.81 in differentiating csPCa and non-csPCa among lesions with a PI-RADS score of 3. Of 30 non-csPCa with PI-RADS score 3, 73% were correctly classified by Textured-DL, and of 13 csPCa lesions with PI-RADS score 3, 85% were correctly classified by Textured-DL. There are still no standardized strategies to predict the risks associated with lesions with a PI-RADS score of 3, but PSA density (PSAD) [13] is commonly used as the reference. Table 4 includes a comparison between PSAD-based classification and Textured-DL. The Textured-DL performed better than the PSAD-based predictions among lesions with a PI-RADS score of 3 by having a high negative predictive value (NPV) while maintaining a high positive predictive value (PPV). This indicated that Textured-DL could potentially serve as an additional tool to predict risks associated with lesions with a PI-RADS score of 3 and further to reduce unnecessary biopsies for these lesions.

The Textured-RF model demonstrated a slightly inferior classification performance compared to Textured-DL. Feeding GLCM directly to deep learning could potentially exploit more texture information than handcrafted texture features from the GLCM. In addition, the proposed model was superior to DCNN and DTL, the state-of-art deep CNNs in the csPCa classification. We believe this can be potentially due to the fact that (1) prostate volume patches must be resized to a fixed size before being fed into DCNN and RESNET, which compromise the scale information of the tumors; and (2) texture describes the tumor heterogeneity, which can be the primary feature of csPCa. Texture information from the GLCM provided the prior knowledge of csPCa for the Textured-DL.

Our study included a few limitations: (1) the patient cohort was based on an MRI dataset at a single academic center. In the future, model evaluation using multi-center MRI datasets can be conducted to test the generalizability of the proposed model. (2) Our study included T2W and ADC for the model. The inclusion of other MRI sequences/components, such as high b-value DWI, dynamic contrast-enhanced (DCE) MRI, and oxygen-enhanced MRI [40], into the model is expected to further improve the PCa classification in the future. (3) The number of patients in the independent testing dataset was not large, particularly for all sub-analyses. Although we observed interesting findings in different tumor locations, types, and PI-RADS categories, larger testing datasets would provide further detailed comparisons between PI-RADS-CLA and Textured-DL. (4) Our study mainly focused on showing the benefit of using a combination of GLCM-based texture information and CNN in the classification of PCa. We believe that other clinical and demographic information, such as PSA, PSA density, age, location of the lesion, patients’ inheritance, BMI, etc., can be combined with our model to improve the performance in the future.

## 5. Conclusions

We proposed a Textured-DL method for the automated PCa classification using 3T mpMRI. The proposed Textured-DL outperformed PI-RADS-CLA in the classification of PCa. The Textured-DL showed superior performance in specificities for the PZ and solitary tumors, compared with PI-RADS-CLA, and demonstrated a sensitivity of 0.85 and a specificity of 0.73 among the lesions with a PI-RADS score of 3.

## Figures and Tables

**Table 1:** Patient and tumor lesion characteristics.

Characteristics		Overall	Train/Validation	Test
Patient Number		402	281	121
csPCa lesions/all lesions		303/466	225/324	78/142
Non-csPCa lesions/all lesions		163/466	99/324	64/142
Age (yr.)		61 (56–66)	61 (56–67)	61 (58–66)
Weight (kg.)		87 (77–95)	87 (77–96)	86 (78–94)
PSA (ng/mL)		8.3 (4.7–8.7)	8.7 (4.7–8.9)	7.4 (4.6–8.7)
Tumor Volume (cm^3^)		1.1 (0.3–1.2)	1.1 (0.3–1.2)	0.9 (0.2–1.1)
GS	False Positive	80	49	31
	3 + 3 = 6	83	50	33
	3 + 4 = 7	168	126	42
	4 + 3 = 7	79	57	22
	>7	56	42	14
PI-RADS score	3	119	76	43
	4	197	138	59
	5	150	110	40
Prostate zone	PZ	364	255	109
	TZ	99	67	32
	AFS	3	2	1
Focality	Solitary	190	137	53
	Multi-focal	276	187	89

For the age, weight, PSA, tumor volume, data are presented as mean (interquartile range). GS = Gleason Score; csPCa = clinically significant prostate cancer; PSA = prostate-specific antigen; PI-RADS = Prostate Imaging Reporting and Data System; PZ = peripheral zone; TZ = transition zone; AFS = anterior fibromuscular stroma.

**Table 2:** Classification performance of Textured-DL on the tumor lesions with different PI-RADS scores.

Lesion Type	csPCa (%)	Non-csPCa (%)	Method	AUC (95% CI)	Sensitivity (95% CI)	Specificity (95% CI)
PI-RADS score 3	13 (30)	30 (70)	Textured-DL	0.81 [0.68–0.94]	0.85 [0.65–1]	0.73 0.58–0.89]
PI-RADS score 4	31 (53)	28 (47)	Textured-DL	0.84 [0.74–0.94]	0.87 [0.75–0.99)	0.71 [0.55–0.88)
PI-RADS score 5	34 (85)	6 (15)	Textured-DL	0.84 [0.66–1]	0.88 [0.77–0.99]	0.67 [0.29–1]

**Table 3:** Performance comparison between PI-RADS-CLA and Textured-DL on the classification of the index tumors with different PSA levels.

Tumor Type	csPCa (%)	Non-csPCa (%)	Method	AUC (95% CI)	*p*-Value	Sensitivity (95% CI)	*p*-Value	Specificity (95% CI)	*p*-Value
All index	77 (84)	15 (16)	PI-RADS-CLA	0.65 [0.52–0.78]	0.32	0.83 [0.75–0.91]	0.25	0.27 [0.04–0.49]	0.18
			Textured-DL	0.73 [0.59–0.88]		0.90 [0.83–0.96]		0.47 [0.21–0.72]	
Index with PSA < 4	10 (77)	3 (23)	PI-RADS-CLA	0.72 [0.41–1.00]	0.06	0.7 [0.42–0.98]	0.32	0.67 [0.13–1.00]	>0.99
			Textured-DL	0.87 [0.58–1.00]		0.9 [0.71–1]		0.67 [0.13–1.00]	
Index with 4 ≤ PSA < 10	53 (87)	8 (13)	PI-RADS-CLA	0.56 [0.37–0.75]	0.35	0.87 [0.78–0.96]	0.03	0.12 [0.00–0.35]	0.56
			Textured-DL	0.43 [0.20–0.65]		0.98 [0.94–1.00]		0.25 [0.00–0.55]	
Index with PSA ≥ 10	14 (78)	4 (22)	PI-RADS-CLA	0.79 [0.58–0.99]	0.17	0.79 [0.57–1.00]	>0.99	0.25 [0.00–0.67]	0.08
			Textured-DL	0.93 [0.80–1.00]		0.79 [0.31–0.83]		1.00 [1.00–1.00]	

**Table 4:** PPV and NPV comparisons between Textured-DL and PSAD-based classification among lesions with a PI-RADS score of 3.

Method	PPV (%)	NPV (%)
Textured-DL	58	92
PSAD ≥ 0.10	29	67
PSAD ≥ 0.15	35	75
PSAD ≥ 0.20	25	68

## Data Availability

The data presented in this study are available on request from the corresponding author. The data are not publicly available due to ethical reasons.

## References

[1] Hoeks C.M.A., Barentsz J.O., Hambrock T., Yakar D., Somford D.M., Heijmink S.W.T.P.J., Scheenen T.W.J., Vos P.C., Huisman H., van Oort I.M. (2011). Prostate Cancer: Multiparametric MR Imaging for Detection, Localization, and Staging. Radiology.

[2] Bjurlin M.A., Carroll P.R., Eggener S., Fulgham P.F., Margolis D.J., Pinto P.A., Rosenkrantz A.B., Rubenstein J.N., Rukstalis D.B., Taneja S.S. (2020). Update of the standard operating procedure on the use of multiparametric magnetic resonance imaging for the diagnosis, staging and management of prostate cancer. J. Urol..

[3] Mottet N., Bellmunt J., Bolla M., Briers E., Cumberbatch M.G., De Santis M., Fossati N., Gross T., Henry A.M., Joniau S. (2017). EAU-ESTRO-SIOG guidelines on prostate cancer. Part 1: Screening, diagnosis, and local treatment with curative intent. Eur. Urol..

[4] Turkbey B., Rosenkrantz A.B., Haider M.A., Padhani A.R., Villeirs G., Macura K.J., Tempany C.M., Choyke P.L., Cornud F., Margolis D.J. (2019). Prostate Imaging Reporting and Data System Version 2.1: 2019 Update of Prostate Imaging Reporting and Data System Version 2. Eur. Urol..

[5] Woo S., Suh C.H., Kim S.Y., Cho J.Y., Kim S.H. (2017). Diagnostic performance of prostate imaging reporting and data system version 2 for detection of prostate cancer: A systematic review and diagnostic meta-analysis. Eur. Urol..

[6] Padhani A.R., Weinreb J., Rosenkrantz A.B., Villeirs G., Turkbey B., Barentsz J. (2019). Prostate imaging-reporting and data system steering committee: PI-RADS v2 status update and future directions. Eur. Urol..

[7] Tewes S., Mokov N., Hartung D., Schick V., Peters I., Schedl P., Pertschy S., Wacker F., Voshage G., Hueper K. (2016). Standardized reporting of prostate MRI: Comparison of the prostate imaging reporting and data system (PI-RADS) version 1 and version 2. PLoS ONE.

[8] Costa D.N., Pedrosa I., Donato F., Roehrborn C.G., Rofsky N.M. (2015). MR imaging–transrectal US fusion for targeted prostate biopsies: Implications for diagnosis and clinical management. Radiographics.

[9] Purysko A.S., Bittencourt L.K., Bullen J.A., Mostardeiro T.R., Herts B.R., Klein E.A. (2017). Accuracy and interobserver agreement for prostate imaging reporting and data system, version 2, for the characterization of lesions identified on multiparametric MRI of the prostate. Am. J. Roentgenol..

[10] Girometti R., Giannarini G., Greco F., Isola M., Cereser L., Como G., Sioletic S., Pizzolitto S., Crestani A., Ficarra V. (2019). Interreader agreement of PI-RADS v. 2 in assessing prostate cancer with multiparametric MRI: A study using whole-mount histology as the standard of reference. J. Magn. Reson. Imaging.

[11] Seo J.W., Shin S.-J., Taik Oh Y., Jung D.C., Cho N.H., Choi Y.D., Park S.Y. (2017). PI-RADS version 2: Detection of clinically significant cancer in patients with biopsy gleason score 6 prostate cancer. Am. J. Roentgenol..

[12] van der Leest M., Cornel E., Israël B., Hendriks R., Padhani A.R., Hoogenboom M., Zamecnik P., Bakker D., Setiasti A.Y., Veltman J. (2019). Head-to-head Comparison of Transrectal Ultrasound-guided Prostate Biopsy Versus Multiparametric Prostate Resonance Imaging with Subsequent Magnetic Resonance-guided Biopsy in Biopsy-naïve Men with Elevated Prostate-specific Antigen: A Large Prospective M. Eur. Urol..

[13] Venderink W., van Luijtelaar A., Bomers J.G.R., van der Leest M., de Kaa C., Barentsz J.O., Sedelaar J.P.M., Fütterer J.J. (2018). Results of targeted biopsy in men with magnetic resonance imaging lesions classified equivocal, likely or highly likely to be clinically significant prostate cancer. Eur. Urol..

[14] Manjunath B.S., Ma W.-Y. (1996). Texture features for browsing and retrieval of image data. IEEE Trans. Pattern Anal. Mach. Intell..

[15] Partio M., Cramariuc B., Gabbouj M., Visa A. Rock texture retrieval using gray level co-occurrence matrix. Proceedings of the 5th Nordic Signal Processing Symposium.

[16] Gatenby R.A., Grove O., Gillies R.J. (2013). Quantitative imaging in cancer evolution and ecology. Radiology.

[17] Avanzo M., Wei L., Stancanello J., Vallières M., Rao A., Morin O., Mattonen S.A., El Naqa I. (2020). Machine and deep learning methods for radiomics. Med. Phys..

[18] Esteva A., Kuprel B., Novoa R.A., Ko J., Swetter S.M., Blau H.M., Thrun S. (2017). Dermatologist-level classification of skin cancer with deep neural networks. Nature.

[19] Liu Y., Yang G., Mirak S.A., Hosseiny M., Azadikhah A., Zhong X., Reiter R.E., Lee Y., Raman S.S., Sung K. (2019). Automatic Prostate Zonal Segmentation Using Fully Convolutional Network With Feature Pyramid Attention. IEEE Access.

[20] Liu H., Li H., Habes M., Li Y., Boimel P., Janopaul-Naylor J., Xiao Y., Ben-Josef E., Fan Y. (2020). Robust Collaborative Clustering of Subjects and Radiomic Features for Cancer Prognosis. IEEE Trans. Biomed. Eng..

[21] Tan J., Gao Y., Liang Z., Cao W., Pomeroy M.J., Huo Y., Li L., Barish M.A., Abbasi A.F., Pickhardt P.J. (2019). 3D-GLCM CNN: A 3-Dimensional Gray-Level Co-Occurrence Matrix-Based CNN Model for Polyp Classification via CT Colonography. IEEE Trans. Med. Imaging.

[22] Heinrich M.P., Jenkinson M., Bhushan M., Matin T., Gleeson F.V., Brady M., Schnabel J.A. (2012). MIND: Modality independent neighbourhood descriptor for multi-modal deformable registration. Med. Image Anal..

[23] Paszke A., Gross S., Chintala S., Chanan G., Yang E., DeVito Z., Lin Z., Desmaison A., Antiga L., Lerer A. Automatic differentiation in pytorch. Proceedings of the 31st Conference on Neural Information Processing Systems (NIPS 2017).

[24] Kingma D.P., Ba J. (2014). Adam: A method for stochastic optimization. arXiv.

[25] Winkel D.J., Tong A., Lou B., Kamen A., Comaniciu D., Disselhorst J.A., Rodriguez-Ruiz A., Huisman H., Szolar D., Shabunin I. (2021). A Novel Deep Learning Based Computer-Aided Diagnosis System Improves the Accuracy and Efficiency of Radiologists in Reading Biparametric Magnetic Resonance Images of the Prostate: Results of a Multireader, Multicase Study. Investig. Radiol..

[26] Bonekamp D., Kohl S., Wiesenfarth M., Schelb P., Radtke J.P., Götz M., Kickingereder P., Yaqubi K., Hitthaler B., Gählert N. (2018). Radiomic machine learning for characterization of prostate lesions with MRI: Comparison to ADC values. Radiology.

[27] der Walt S., Schönberger J.L., Nunez-Iglesias J., Boulogne F., Warner J.D., Yager N., Gouillart E., Yu T. (2014). Scikit-image: Image processing in Python. PeerJ.

[28] Ho T.K. Random decision forests. Proceedings of the 3rd International Conference on Document Analysis and Recognition.

[29] Pedregosa F., Varoquaux G., Gramfort A., Michel V., Thirion B., Grisel O., Blondel M., Prettenhofer P., Weiss R., Dubourg V. (2011). Scikit-learn: Machine learning in Python. J. Mach. Learn. Res..

[30] Zhong X., Cao R., Shakeri S., Scalzo F., Lee Y., Enzmann D.R., Wu H.H., Raman S.S., Sung K. (2019). Deep transfer learning-based prostate cancer classification using 3 Tesla multi-parametric MRI. Abdom. Radiol..

[31] He K., Zhang X., Ren S., Sun J. Deep residual learning for image recognition. Proceedings of the IEEE Conference on Computer Vision and Pattern Recognition.

[32] Song Y., Zhang Y.-D., Yan X., Liu H., Zhou M., Hu B., Yang G. (2018). Computer-aided diagnosis of prostate cancer using a deep convolutional neural network from multiparametric MRI. J. Magn. Reson. Imaging.

[33] Simonyan K., Zisserman A. (2014). Very deep convolutional networks for large-scale image recognition. arXiv.

[34] Weinreb J.C., Barentsz J.O., Choyke P.L., Cornud F., Haider M.A., Macura K.J., Margolis D., Schnall M.D., Shtern F., Tempany C.M. (2016). PI-RADS prostate imaging–reporting and data system: 2015, version 2. Eur. Urol..

[35] Agresti A., Coull B.A. (1998). Approximate is Better than “Exact” for Interval Estimation of Binomial Proportions. Am. Stat..

[36] Fluss R., Faraggi D., Reiser B. (2005). Estimation of the Youden Index and its associated cutoff point. Biom. J. J. Math. Methods Biosci..

[37] DeLong E.R., DeLong D.M., Clarke-Pearson D.L. (1988). Comparing the areas under two or more correlated receiver operating characteristic curves: A nonparametric approach. Biometrics.

[38] Eliasziw M., Donner A. (1991). Application of the McNemar test to non-independent matched pair data. Stat. Med..

[39] Gaur S., Lay N., Harmon S.A., Doddakashi S., Mehralivand S., Argun B., Barrett T., Bednarova S., Girometti R., Karaarslan E. (2018). Can computer-aided diagnosis assist in the identification of prostate cancer on prostate MRI? a multi-center, multi-reader investigation. Oncotarget.

[40] Zhou H., Hallac R.R., Yuan Q., Ding Y., Zhang Z., Xie X.-J., Francis F., Roehrborn C.G., Sims R.D., Costa D.N. (2017). Incorporating oxygen-enhanced MRI into multi-parametric assessment of human prostate cancer. Diagnostics.

